# Opportunities in Cancer Therapies: Deciphering the Role of Cancer Stem Cells in Tumour Repopulation

**DOI:** 10.3390/ijms242417258

**Published:** 2023-12-08

**Authors:** Loredana G. Marcu, Mikaela Dell’Oro, Eva Bezak

**Affiliations:** 1 UniSA Allied Health & Human Performance, University of South Australia, Adelaide, SA 5001, Australia; eva.bezak@unisa.edu.au; 2 Faculty of Informatics and Science, University of Oradea, 410087 Oradea, Romania; 3 Australian Centre for Quantitative Imaging, School of Medicine, The University of Western Australia, Perth, WA 6009, Australia; mikaela.delloro@uwa.edu.au; 4 Faculty of Chemistry & Physics, University of Adelaide, Adelaide, SA 5000, Australia

**Keywords:** tumour regrowth, repopulation mechanisms, radiotherapy, chemotherapy, imaging, nanotechnology

## Abstract

Tumour repopulation during treatment is a well acknowledged yet still challenging aspect of cancer management. The latest research results show clear evidence towards the existence of cancer stem cells (CSCs) that are responsible for tumour repopulation, dissemination, and distant metastases in most solid cancers. Cancer stem cell quiescence and the loss of asymmetrical division are two powerful mechanisms behind repopulation. Another important aspect in the context of cancer stem cells is cell plasticity, which was shown to be triggered during fractionated radiotherapy, leading to cell dedifferentiation and thus reactivation of stem-like properties. Repopulation during treatment is not limited to radiotherapy, as there is clinical proof for repopulation mechanisms to be activated through other conventional treatment techniques, such as chemotherapy. The dynamic nature of stem-like cancer cells often elicits resistance to treatment by escaping drug-induced cell death. The aims of this scoping review are (1) to describe the main mechanisms used by cancer stem cells to initiate tumour repopulation during therapy; (2) to present clinical evidence for tumour repopulation during radio- and chemotherapy; (3) to illustrate current trends in the identification of CSCs using specific imaging techniques; and (4) to highlight novel technologies that show potential in the eradication of CSCs.

## 1. Cancer Stem Cell Properties

Cancer stem cells are recognised as an important cellular category that is at the top of the hierarchical scale, presenting similar biological properties and expression profiles to normal stem cells [1]. While the origins of cancer stem cells in solid tumours are not fully elucidated, it is hypothesised that they arise from either differentiated tumour cells or from organ-specific adult stem cells [2].

### 1.1. Biological Characteristics of CSCs

Generally, cancer stem cells (CSCs) account for a very small percentage of the cells that constitute a malignant solid tumour [3]. The fraction of CSCs within a tumour was shown to be dependent on the neoplasm, but also on the in vivo assays and biomarkers employed for their identification [4]. An interesting observation derived from in vitro studies on CSCs is related to their heterogeneity [5]. Research undertaken on both HPV-positive and HPV-negative head and neck carcinoma cell lines revealed the existence of a diversity of CSCs that differed in their biological properties and response to therapy, explaining the more responsive behaviour of HPV-positive tumours [5]. This finding demonstrates than not only the absolute percentage of CSCs but also their phenotype dictate treatment outcome, showing the complexity that surrounds CSC identification and targeting. The link between oncogenic viruses and CSCs was also investigated in gastric cancers associated with the Epstein–Barr virus [6,7]. Epstein–Barr virus-encoded miRNAS were shown to have a strong impact on the maintenance of stemness through the regulation of epithelial–mesenchymal transition. Recent studies on gastric cancers identified a unique CSC marker (CD44v6/v9) for Epstein–Barr virus-associated carcinomas that might serve as a potential target for this subtype of gastric cancer [7].

Despite their low percentage, the biological properties specific to CSCs are powerful tools that influence the fate of the tumour. Among their biological features, pluripotency is perhaps one of the most notable ones, whereby CSCs are capable of unlimited self-renewal by maintaining an undifferentiated state, also being able to create the entire heterogeneous lineage of the original tumour through differentiation [8]. Stemness is maintained by cellular plasticity—a property that promotes tumour heterogeneity and CSC generation.

### 1.2. CSC and Hypoxia

A particular property of cancer stem cells is their preference for residing in specific microenvironmental niches, such as hypoxic ones, in order to preserve their functionality and stem-like properties [9]. Studies showed that the hypoxia inducible factor (HIF) plays a key role in the niche mechanism, being implicated in CSC survival and proliferation under hypoxic conditions as well as in the angiogenic switch activation [10]. Knowing the challenge of treatment resistance caused by the presence of hypoxia, tumour control is heavily impacted by the increased expression of HIF found in these hypoxic niches.

### 1.3. CSC Repopulation and Resistance to Therapy

CSCs are also characterised by quiescence, a state that fosters prolongation of their life span, preservation of cellular functions, and protection from therapeutic stress [11]. CSC quiescence was shown to induce treatment resistance and tumour recurrence while also promoting metastases. Another mechanism adopted by CSCs is cellular senescence, which was shown to be responsible for genetic reprogramming and the activation of stemness, contributing to tumour progression and distant spread [11].

All the above-mentioned CSC properties have important clinical implications, as stem-like cancer cells have been shown to be more resistant to treatment than their non-stem counterparts, often leading to treatment failure [12]. One of the reasons for poor treatment outcome is credited to tumour repopulation during therapy. Today it is known that repopulation during treatment is not limited to radiotherapy, as there is clinical proof of repopulation mechanisms being activated through other conventional treatment techniques, such as chemotherapy. The dynamic nature of stem-like cancer cells often elicits resistance to treatment by escaping drug-induced cell death.

The mechanisms behind repopulation are complex and most are attributed to the presence of CSCs [13,14,15]. According to previous research, CSCs adopt various repopulation mechanisms in response to the effect of chemo/radiotherapy. Among them, cell recruitment, shortening of cell cycle duration, and the loss of asymmetrical division are the most commonly investigated and agreed upon mechanisms [13,14,15,16]. Quiescence or dormancy, as previously mentioned, is a state outside the cell cycle where CSCs reside to prolong their lifespan and preserve functionality. However, a situation might arise, such as cellular eradication, which triggers the re-entry of CSCs into the cell cycle through the process of cell recruitment. Once in the mitotic cycle, stem cells can further activate repopulation mechanisms by shortening the length of their passage through the cycle, thus promoting faster cell turnover. Perhaps the most efficient repopulation mechanism is the symmetrical division of CSCs (i.e., the creation of two cancer stem cells) which is acquired through the loss of asymmetrical division (i.e., the creation of a stem and a differentiated cell) [15,17].

The goals of this scoping review are to gather evidence towards the role played by cancer stem cells in tumour repopulation during cancer treatment, and to identify techniques to either counteract these mechanisms or to overcome repopulation by direct targeting of cancer stem cells.

## 2. Repopulation during Radiotherapy

### 2.1. Cancer Stem Cells and Their Role in Repopulation during Radiotherapy

Tumour repopulation after radiotherapy remains a significant challenge in cancer therapy. Despite advancements in photon radiation therapy techniques, such as intensity modulated radiotherapy (IMRT) or volumetric modulated arc radiotherapy (VMAT), the recurrence of tumours post-treatment remains an issue for many cancers, including head and neck, breast, colorectal, prostate, and hepatocellular cancers [18,19,20,21].

As discussed elsewhere in this paper, once again, clonogenic stem cells have emerged in the literature as primary drivers in the process of tumour repopulation that ultimately lead to treatment failure [22,23]. Because of CSCs’ propensity for renewal, differentiation into various cell types, as well as recruitment of differentiated cells and treatment resistance, they drive tumour repopulation during and post radiation therapy. In other words, the ongoing division of clonogenic cancer stem cells has been shown to be the cause of tumour regrowth in, for example, intestinal, breast, and brain tumours [24].

Similarly to chemotherapy, CSCs are often more resistant to radiation therapy compared to the bulk of tumour cells [25]. This CSC resistance is attributed to various mechanisms, including enhanced DNA repair capacity, quiescence, higher expression of drug efflux pumps, resting in tumour hypoxic regions, etc., which collectively contribute to their survival and capacity to initiate tumour repopulation and regrowth [26]. This phenomenon is a significant obstacle to achieving durable therapy responses in cancer patients.

In addition to radiation resistance, another key characteristic of CSCs is their ability to be recruited from the quiescent state following the irradiation damage and re-enter a standard cell cycle, thus initiating cell proliferation and differentiation, leading to the regrowth of the tumour. Research has shown that about one third of CSCs in glioma and breast cancer cell lines remain quiescent but become active in the cell cycle following radiation treatment [27,28]. The work of Bao et al. [29], conducted in animal models using primary human gliomas, showed that CD133 (Prominin-1), a marker for both neural stem cells and brain cancer stem cells, was not only enriched after irradiation but also capable of initiating xenografts from as few as 500 cells. The authors conclude that CD133-positive tumour cells represent the cellular population responsible for glioma radioresistance and potentially tumour recurrence after radiation therapy [29].

CSCs can also promote tumour heterogeneity by generating different types of CSCs through reversible transformations between stem and non-stem cells [30,31]. This heterogeneity further increases treatment resistance and contributes to cell survival and subsequent repopulation. Preclinical work by Lagadec et al. reports that upregulation of the embryonic transcription factors (such as Sox2, Oct4, Klf4, and Nanog) induced by radiation promotes nontumorigenic cancer cells to acquire CSC-like features [32].

Tumour repopulation itself after radiation therapy can be attributed to several processes. One key factor is the stimulation of surviving cancer cells through a phenomenon known as radiation-induced cytokine release. The irradiated tumour microenvironment can trigger the secretion of growth factors, such as TGF-β and VEGF, which promote the survival and proliferation of cancer cells [33]. Moreover, the radiation-induced DNA damage repair can lead to genetic mutations and clonal expansion of radioresistant cancer cells. This is further compounded by the fact that CSCs have pro-survival pathways that are upregulated and protect these cells from cell death, resulting in CSCs being resistant to radiation damage [34].

### 2.2. The Effect of Radiotherapy on CSCs

Fractionated radiation therapy, while necessary to mitigate the damage to healthy tissues, is selective in its cell kill. It eliminates, by and large, the differentiated tumour cells and enables the preferential survival of the most radiation therapy resistant and more tumorigenic CSCs, thus generating a so-called radiation-induced CSC phenotype [21,35,36]. This in turn enhances the malignancy of residual tumours. In breast cancer, the proportion of CSCs increases significantly after ionizing radiation, leading to enhanced proliferation, which accelerates tumour repopulation [32,37]. The cell line experiments by Lagadec et al. showed that these radiation-induced CSC phenotypes often exhibit enhanced sphere-forming and tumorigenic abilities. This means that the number of tumorigenic cells increases after treatment, potentially leading to a more rapid tumour recurrence and increased aggressiveness that can lead to metastatic disease [32].

Additionally, the selective killing that enables the radiation induced CSC phenotype is particularly relevant in cases where the overall treatment time is prolonged, which has been associated with a decrease in tumour control rates in certain types of cancers. A seminal study by Withers et al. identified rapid tumour regrowth during extensions of radiotherapy treatment from ~5–8 weeks in almost 500 patients with oropharyngeal cancer. They concluded that for rapidly proliferating cancers such as head and neck, radiotherapy should be completed within the shortest feasible time due to accelerated repopulation of CSCs that occurs while the tumour is still regressing. In view of this, the recommendation is that a delay in treatment initiation is preferable to delays during radiotherapy [18].

### 2.3. Strategies to Inhibit Tumour Regrowth after Radiotherapy and Future Perspectives

Fast tumour repopulation owing to CSCs gives justification for the implementation of shorter treatment courses and hypofractionation regimens, such as stereotactic ablative radiotherapy (SABR) or hyperfractionated regimens in head and neck cancers, to avoid quick repopulation between two subsequent doses. Where plausible, dose escalations also help to counteract the processes supporting tumour repopulation [38]. Additionally, combining radiation therapy with targeted therapies, such as EGFR inhibitors or immune checkpoint inhibitors, can enhance the radiosensitivity of cancer cells and suppress repopulation.

Other options include the use of protons and heavy ions that generate more complex and direct DNA radiation-induced damage, which may be more independent of oxygen status, overcoming the resistance of hypoxic cells. Chiblak et al. showed that primary human glioma stem cells with resistance to photon therapy could be made sensitive to treatment with carbon ions due to their compromised ability to repair DNA double-strand breaks caused by carbon ion irradiation [39].

The interplay between CSCs and tumour repopulation post-radiotherapy is a complex and multifaceted phenomenon. The large heterogeneity in CSC radiosensitivity which leads to both inter- and intra-patient variability makes a one-size-fits-all treatment approach challenging. Understanding the biology and resistance mechanisms of these cells is critical for advancing cancer treatment strategies. Future research should focus on elucidating the molecular pathways involved and identifying innovative approaches to target clonogenic stem cells, ultimately improving the success rates of radiotherapy as well as combined therapies, thus reducing the risk of tumour recurrence.

Efforts to mitigate tumour repopulation after radiotherapy have led to investigations into strategies that specifically target clonogenic stem cells. These strategies encompass the development of CSC-specific therapies, such as CSC-targeting drugs and combination treatments. While promising, challenges remain in effectively eradicating or sensitizing clonogenic stem cells to radiotherapy.

## 3. Repopulation during Chemotherapy

### 3.1. Cancer Stem Cells and Their Role in Repopulation during Chemotherapy

While the role of cancer stem cells in the process of tumour repopulation during radiotherapy is well researched and supported by experimental evidence, accelerated repopulation during the course of chemotherapy is often overlooked, despite similar biological triggers—such as loss of cancer cells—being involved in both types of cancer therapy [40].

**Figure 1:**
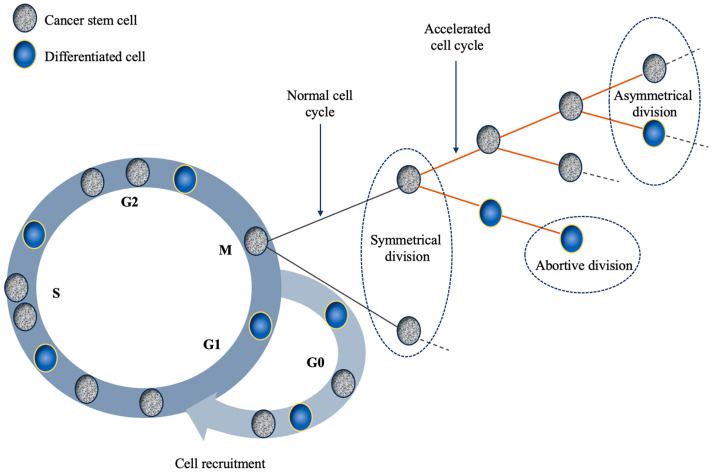
Schematic representation of the main mechanisms responsible for tumour repopulation during therapy. The four main mechanisms discussed in the text that are depicted in this illustration are the following: (1) cell recruitment: G_0_ cells re-enter the cell cycle; (2) symmetrical division: a stem cell divides into two stem cells; (3) abortive division: non-stem cells exhibit finite proliferative ability; (4) accelerated division: stem cells shorten the duration of their mitotic cycle.

In silico studies looking into the mechanisms behind repopulation during drug-based therapies concluded that chemotherapy could trigger tumour regrowth through cell recruitment into the mitotic cycle [41]. This is a potent biological mechanism considering the fact the quiescent cells are not affected by chemotherapy (being non-proliferative), thus the pool of resting cells in the G_0_ phase is large and viable (Figure 1). Although the onset of tumour repopulation during chemotherapy is a poorly researched factor, the limited experimental data suggests that this process might occur at various degrees and intervals after drug administration, owing to different recruitment rates for each particular tumour type [40]. In silico data showed that early onset of repopulation (during the first week of chemotherapy) has a more powerful effect on the outcome than late onset (during the second half of therapy), an expected result as long as the rate of recruitment remains constant [41]. Nevertheless, treatment outcome is strongly influenced by the recruitment rate, which interplays with the onset of repopulation, thus dictating together the fate of cancer cells [41].

Another mechanism identified as a contributor towards repopulation is abortive division [14], but with a less powerful impact than cell recruitment as it affects differentiated cells with finite proliferating ability (hence the term ‘abortive division’) which only have a short-term, transitory bearing on tumour population [42].

A rather paradoxical phenomenon concerning tumour repopulation and treatment resistance is facilitated by cellular senescence. Senescence, an essential tumour suppressor mechanism, is a cellular response to various stress-inducing stimuli (such as radio/chemotherapy) whereby cells lose their proliferative ability [43]. At the same time, senescent cells can spontaneously evade their condition and regain their proliferative ability re-entering the cell cycle, which is clear proof of cellular plasticity [44]. Thus, instead of tumour suppression, treatment-induced senescence can, depending on microenvironmental factors, lead to an opposite effect, i.e., repopulation and resistance to therapy.

Drug resistance is a very common factor hindering the success of chemotherapy, and it was shown to be linked to tumour repopulation during treatment [45]. Resistance to chemotherapy is a cumulative process that ultimately overpowers tumour regression leading to poor treatment outcome. Therefore, management of drug resistance is one of the key requirements to avoid cancer cell survival that could lead to repopulation during cycles of chemotherapy. Lately, the tumour microenvironment was shown to play an important role in therapy resistance, due to its potential to dictate the maintenance of CSCs through the release of cytokines, growth factors, and other cellular components to promote stemness [46]. While the targeting of CSCs is a critical factor for overcoming repopulation and resistance to treatment, the complex tumour microenvironment together with its pathways to promote stemness require well-targeted, combined approaches to inhibit tumour proliferation.

Recent studies on human colon cancer stem cells highlighted the drug-tolerant characteristics of these CSCs (called persister cells), i.e., their ability to escape cell death induced by a wide range of chemotherapeutic agents [47]. The study enabled the investigation of in vivo CSC dynamics through the tracking of LGR5+ cells with stem-like properties. A quiescent subset of cells expressing the p27 protein has been identified, an expression that is a feature of persister cells. Chemotherapy was shown to disrupt the dormancy of these cells, triggering them into the cell cycle, and thus into the regrowth process. Similar chemotherapy-stimulated transition into the proliferative state was evidenced in previously quiescent glioblastoma cancer stem cells in mouse models [48]. The glioblastoma study confirmed the unique feature of cancer stem cells and the CSC-specific relevance of functional markers in the context of tumour diversity.

### 3.2. Clinical Evidence Concerning the Impact of CSC Repopulation on Treatment Outcome

When employing non-conventional chemotherapy regimens to overcome the challenges posed by accelerated repopulation, the results of randomised clinical trials are perhaps the most convincing indication towards tumour regrowth during therapy. Several trials designed for various tumour sites have been conducted to evaluate the effect of non-traditional treatment schedules on tumour response [49,50,51]. The response of unresectable non-small-cell lung cancers (NSCLC) to accelerated treatment regimens was heavily investigated owing to the known potential of accelerated schedules to overcome repopulation during therapy in tumours with short cell doubling time. A phase I trial designed for inoperable NSCLC patients aimed to evaluate the maximum tolerated dose (MTD) of accelerated hypofractionated radiotherapy (3 Gy/fraction, over 5 weeks) concurrently with vinorelbine and carboplatin, in a dose escalation setting (69 Gy was regarded as the MTD) [49]. The short-term response rate was 84.6%, with a 1-year median progression-free survival of 49.4%, showing good local tumour control for this patient group, and thus warranting further investigation.

Maguire et al. reported the results of a randomised phase II trial (SOCCAR trial) that compared sequential with concurrent cisplatin–vinorelbine-based chemotherapy and radical hypofractionated radiotherapy (over 4 weeks) in nonresectable NSCLC patients to assess their effectiveness in minimising accelerated repopulation [50]. The results showed slightly higher 2-year overall survival with the concurrent chemotherapy regimen (50% vs. 46%), with no difference in toxicity between the two arms, concluding that concurrent drug-radiotherapy is a safe and efficient way to counteract the effect of tumour repopulation during treatment.

Since overall treatment time plays a critical role in determining the success of therapy in fast growing tumours, a multicentre, prospective, randomised clinical trial was designed for NSCLC patients, aiming to investigate the effect of accelerated postoperative radiochemotherapy vs. conventionally fractionated therapy in increasing local tumour control by administering seven fractions of two Gy doses per week of either photons or protons [51]. Using this accelerated treatment regimen, it was postulated that the 3-year local tumour control will increase from 70% to 85%. The final results of this trial have not been published so far.

In invasive bladder cancers with fast repopulation of cancer stem cells, Panteliadou et al. conducted a trial employing an accelerated hypofractionation (HypoARC) with 15 fractions of 3.4 Gy to the bladder, together with amifostine to alleviate radiation-induced side effects [52]. The treatment outcome showed an 86.6% complete response rate and 56% 3-year local control, the results being comparable with previous reports. While this schedule seemed safe, particularly due to the administration of amifostine, local tumour control did not present notable differences from more conventional schedules, highlighting the fact that changes in treatment regimens are not always sufficient to overcome tumour repopulation.

### 3.3. CSC Sensitivity Assay-Guided Chemotherapy

In view of the above, the development of CSC sensitivity assays to guide chemotherapy delivery would allow for treatment personalisation in a variety of cancer types. However, there are several challenges in developing efficient procedures, from tissue sample collection to the length of the test, impacting the clinical implementation [53]. To date, the only US-certified and accredited CSC sensitivity assay is ChemoID, developed to examine the response of CSCs derived from patient samples to conventional chemotherapeutic agents [54,55].

Perhaps one of the most investigated tumours in the context of cancer stem cell assay-guided chemotherapy is glioblastoma, owing to its aggressive nature and resistance to therapy [54,55,56]. Prominin-1, or CD133, is a widely explored CSC biomarker associated with normal neural stem cells, and thus is commonly used in glioblastomas, together with other CSC markers such as CD24, CD44, CXCR4, Oct3/4, and Nanog. ChemoID was employed to evaluate the correlation between drug efficiency (temozolomide) and CSC positivity using tissue samples collected from a cohort of 41 patients treated with standard temozolomide radiotherapy and to assess this association with tumour recurrence [54]. Patients with a positive CSC test (>40% in vitro cell kill) had a median recurrence time of 20 months compared to 3 months for patients with negative CSC tests. The same assay was used in a prospective glioblastoma study to guide primary treatment after analysing the biopsies originating from 14 patients for the most effective drug therapy [55]. Despite unfavourable outcome predictors, patients treated with ChemoID guidance had an 86% overall response rate and a 13.3-month median overall survival compared to the historical median of 9.1 months [55]. These preliminary results were just confirmed by the results of a randomised clinical trial on patients with recurrent glioblastoma, showing the potential of CSC drug response assay to assist with treatment stratification by identifying patients that would benefit from a specific chemotherapy [56].

Another candidate for ChemoID was platinum-resistant ovarian cancer in an attempt to improve treatment outcome in 78 patients affected by the third relapse of their cancer [57]. Beside the choice of drug, the assay assisted in decisions regarding dose escalation/reduction to optimise tumour response and minimise normal tissue toxicity. Patients treated with high cell kill chemotherapy based on the CSC drug response assay showed improved outcomes compared to those with negative CSC tests (15 vs. 6 months overall survival) or when compared to historical cohorts (15 vs. 8.9 months overall survival) [57]. Since this assay demonstrated efficiency in aggressive cancers, it could be considered a tool for preventing recurrence in high-risk patients and for guiding chemotherapy when multiple drugs are clinically available for a particular cancer.

### 3.4. CSC-Targeting Agents and Mechanisms

As previously stated, non-conventional chemo/radiotherapy regimens have often been employed in clinics to overcome tumour repopulation. However, their limited or tumour-specific success motivated the investigation of other pathways to suppress resistance to treatment caused by CSC repopulation. The Hedgehog pathway is known to play a critical role in several cellular functions, including signal transmission from the membrane to the nucleus for normal tissue development and regeneration. Studies in gastric cancers have identified a subgroup of CSCs as being responsible for aberrant activation in the Hedgehog signalling pathway, leading to chemotherapy resistance [58,59]. Gastric cancers positive for CD44 showed resistance to several traditional drugs, which was reversed in vitro through the inhibition of the Hedgehog signal using smoothened shRNA or vismodegib. Tumour samples from a phase II clinical trial of gastric patients showed a correlation between high CD44 expression and decreased survival, while the addition of vismodegib to chemotherapy to the group presenting high CD44 expression was associated with improved outcome [58].

Another protein identified to interplay with CSCs in a gastric cancer subtype (diffuse gastric cancer) is RhoA, a member of the Rho GTPase family, being involved in varied cellular functions such as proliferation and survival [59]. Based on the increased activity of RhoA found in gastric cancers, Yoon et al. suggested that RhoA is likely to cause resistance to chemotherapy through the promotion of CSCs [59]. To prove their theory, xenografts were grown to be treated with cisplatin and RhoA pathway inhibitors, showing a significant decrease in cells expressing CD44. The study shows not only the complexity of targeting CSCs but their different phenotype that requires tumour-based personalised approaches.

Efficient eradication of cancer stem cells requires their identification and precise targeting [60]. Metformin, a repurposed diabetes drug, was found to be a modulator of cellular growth and metabolism, inducing AMPK activation and inhibition of tumour development [61]. It has been identified as an effective, though selective, CSC-targeting agent in certain cancers, including colorectal and ovarian cancers [61,62]. The results of a nonrandomised phase II clinical trial of metformin combined with platinum-based chemotherapy for patients with advanced epithelial ovarian cancer showed a positive outcome on ALDH+ cancers [62]. The administration of metformin resulted in a 2.4-fold reduction of CSCs in ovarian cancers as well as an alteration of mesenchymal stem cells which in turn eliminated resistance to platinum. Currently, there are 89 active or recruiting clinical trials evaluating the effect of metformin on treatment outcome in a variety of cancers [63].

Blood cancers, such as acute myeloid leukaemia, are also driven by cancer stem cells. This aspect makes them resistant to conventional chemotherapy, which usually leads to recurrence of disease. Preclinical studies have identified possible targets in acute myeloid leukaemia such as cells expressing CD70 and the CD70/CD27 signalling pathway, which were further tested in a phase I/II clinical trial using cusatuzumab—a human αCD70 monoclonal antibody exhibiting enhanced toxicity on leukemic stem cells with promising results [64].

Another approach to interfering with tumour repopulation is to target senescence-induced cancer stem cells. As explained above, senescence is a double-edged sword, being a potent tumour suppressor under certain conditions while promoting cancer stem cells via cellular plasticity in other circumstances [44]. Through the latter process, senescent cells acquire a senescence-associated secretory phenotype (SASP) with pro-tumorigenic effects. Thus, targeting SASP could interfere with the initiation of senescence-induced CSCs. In view of this, a number of preclinical studies have investigated SASP-targeting pathways in combination with chemotherapeutic agents to assess the effect on CSC suppression and inhibition of chemoresistance, pointing towards a feasible clinical application [65,66].

## 4. Future Avenues in CSC Management

### 4.1. Imaging

The capacity to observe populations of cancer stem cells (CSC) plays a crucial role in assessing the immediate efficacy of therapeutic measures and making necessary adjustments to enhance treatment results. Utilising quantitative imaging methods for identification and ongoing monitoring holds the promise of real-time tracking of these populations and gauging treatment responses, ultimately facilitating treatment optimisation [67]. Presently, various imaging techniques are employed, including radionuclide imaging methods such as positron emission tomography (PET) and single-photon emission computed tomography (SPECT), magnetic resonance imaging (MRI), intravital imaging, bioluminescence imaging, as well as different forms of fluorescence imaging, including fluorescence-mediated tomography and near-infrared fluorescence reflectance imaging. Researchers are actively exploring advancements in these imaging technologies to unravel the intricate biological aspects of CSC behaviour, with promising implications for clinical applications [68]. Noteworthy reviews have focussed on the application of functional MRI and developments in nuclear medicine imaging techniques, PET, and SPECT, for the identification and tracking of endogenous CSCs [60,69].

PET quantitatively identifies high-energy γ-rays originating from a subject injected with positron-emitting isotopes or isotope-tagged molecular probes. It boasts remarkable sensitivity, offers non-invasive capabilities, enables real-time in vivo monitoring, and functions independently of the emission source’s depth. Nonetheless, at present, there is no PET technology capable of achieving single-cell resolution for the detection of CSC.

Multiple research teams have reported on various innovative imaging agents and tracers designed for CD133, aimed at facilitating the non-invasive detection of CSCs in different types of malignancies. A study by Hu et al. [70] harnessed CM-2, a stabilised variant of the CM peptide that specifically targets CD133, and created a stable tracer named [64Cu]Cu-CM-2 for PET imaging. They assessed its potential for imaging CD133-expressing tissues within a mouse bearing Huh-7 tumours, revealing its accumulation and retention in the tumour regions. In an earlier investigation, Jin et al. [71] explored the possibility of radioimmuno-targeting CSCs using PET or SPECT. They delved into the feasibility of in vivo radionuclide imaging of CSCs by employing Iodine-125-labelled ANC9C5, an anti-human CD133 antibody, in colon carcinoma xenografts. While they did not achieve an ideal biodistribution profile, autoradiography demonstrated overlapping distribution of 125I-labelled ANC9C5 with CD133 immunohistochemistry expression in many regions. Efforts to enhance accuracy, the biocompatibility of tracers, as well as advancements in techniques, imaging resolution, and contrast are ongoing. For instance, a recent development includes a novel and highly specific 68Ga peptide-based PET imaging agent designed for CD133 imaging in colorectal cancer by Liu et al. [72].

MRI is a non-invasive imaging method renowned for its robustness and high spatial resolution. One notable advantage of MRI is its independence from radioactive isotopes, making it particularly suitable for longitudinal studies. To date, various contrast agents, including superparamagnetic or paramagnetic nanoparticles, ultrasmall superparamagnetic nanoparticles, fluorine, gadolinium, and specific reporter genes, have been employed for CSC imaging.

Among these, ultrasmall superparamagnetic iron oxide (SPIO) or superparamagnetic iron oxide labels are commonly utilised for cell tracking via magnetic resonance. Notably, SPIO has yielded promising outcomes, successfully detecting and monitoring transplanted glioblastoma CSCs in vitro [73]. In a recent investigation by Sun et al. [74], researchers explored the use of SPIO-loaded glioblastoma stem cells to enhance radiation therapy and act as a contrast agent for imaging purposes. Their findings showcased heightened DNA damage in glioblastoma cells when combined with radiation therapy. Further development and testing may lead to their incorporation as theranostic agents, serving dual roles as an MRI-based contrast agent and a radiosensitiser, with potential applications in both treatment and imaging.

### 4.2. Theranostics

Theranostics is a rapidly evolving field, with several integrated approaches and techniques already being implemented in clinical practice. There is a growing body of literature highlighting the use of alpha particles, including astatine-211, actinium-225 (225Ac), lead-212 (212Pb), and thorium-227, all of which induce challenging-to-repair, clustered DNA double-strand breaks. In clinical settings, 177Lu remains the prevailing radioisotope of choice. Beyond safety data on routinely used alpha-emitting radioisotopes, such as radium-223 (223Ra), clinical trials involving 212Pb-DOTAMTATE have exhibited promising safety profiles. The ongoing emphasis on developing new radiopharmaceuticals and the recent advancements in receptor-targeted strategies present an exciting outlook for the theranostics of CSCs.

**Figure 2:**
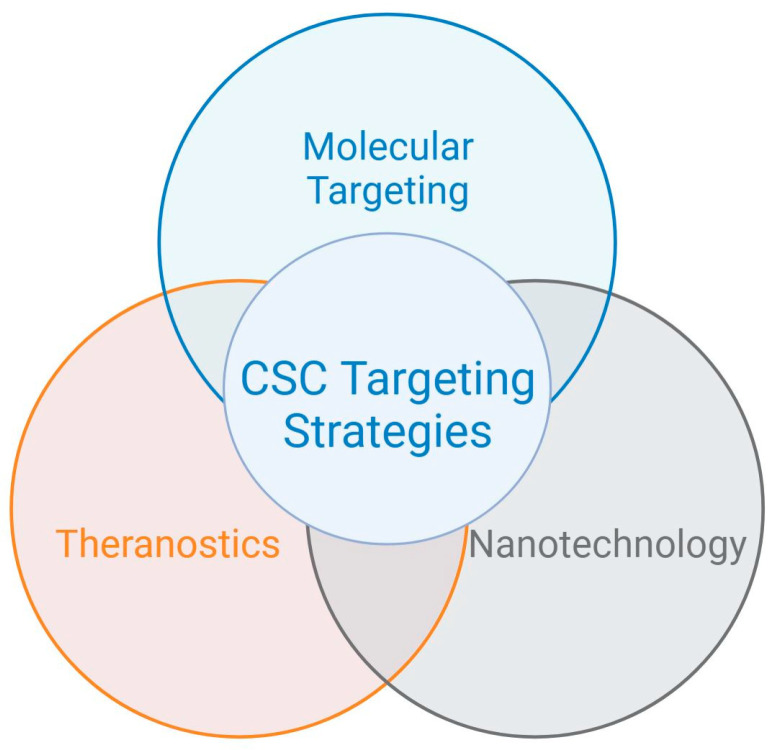
Diagram illustrating the overlap of future CSC management techniques. Initiatives have commenced to address CSC by employing targeting cell-surface markers or monoclonal antibodies, coupled with progress in the delivery of nanoparticles and radiopharmaceuticals.

Recently, new therapeutic strategies such as CSC-targeted therapies and combination treatments have been employed to investigate the effective treatment of CSCs (Figure 2). One of the most promising methods involves the use of monoclonal antibodies (mAbs). Several noteworthy preclinical investigations in the field of theranostics encompass the use of 125I/225At-labelled anti-CXCR4 monoclonal antibody for acute myeloid leukaemia [75], 177Lu-labelled anti-EGFR mAb for breast cancer [76], 111In-NLS-CSL360 for leukaemia [77], 188Re-6D2 for melanoma [78], and 111In-DTPA-CD166tp-G18C for colorectal cancer [79]. While these studies are currently in the preclinical phase, the ongoing emphasis on developing novel radiopharmaceuticals and the recent advancements in receptor-targeted strategies present an exciting outlook for the application of theranostics in CSCs.

Numerous potential markers exist for identifying CSCs across various types of cancers [80]. These CSCs express a range of cell-surface markers, including CD133, CD44, and CD24, as well as ABC transporters such as ABCG2 and ABCB5. These biomarkers are essential for the isolation and analysis of the biological and physiological characteristics specific to CSC populations. Nevertheless, it is important to note that these biomarkers are not exclusive to CSCs; they also mark populations enriched with CSCs and may be present in normal tissue stem cells, rendering them unsuitable for targeted strategies [81].

Efforts have been undertaken to utilise CD133 as a target in cancer theranostics. In a study by Sun et al. [82], an RNA aptamer was linked to cationic liposomes and loaded with siRNA and paclitaxel. The delivery of siRNA and paclitaxel to glioma stem cells induced apoptosis, while increasing the differentiation of CD133+ glioma stem cells into non-stem cells improved the overall survival rate in mice bearing glioma tumours.

The CXCR4 is recognised for its elevated expression in CSCs across more than 20 human tumour types, and it is associated with tumorigenicity, angiogenesis, invasion, and resistance to chemotherapy [83]. High levels of CXCR4 expression are significantly linked to distant metastasis, poor overall survival, and reduced disease-free survival in various cancer types, including breast, prostate, pancreatic, and lung cancers, as well as lymphoma and leukaemia, making it an independent prognostic factor in these diseases [84].

The clinical application of 68Ga-pentixafor for CXCR4-directed PET imaging and the investigation of radionuclide therapies involving 177Lu- and 90Y-Pentixather have shown promising potential in theranostics. Additionally, a newly developed CXCR4 antagonist, PRX177561, has demonstrated a significant reduction in glioblastoma tumour growth and the potential to enhance the effects of anticancer chemotherapy and radiotherapy. In addition, PRX177561 has extended disease-free survival and overall survival in mice carrying orthotopic brain xenografts [85].

Further research is essential to validate and explore the therapeutic potential of these markers and factors in various cancer types.

### 4.3. Nanotheranostics

Nanotheranostics represents an innovative fusion of therapeutic and diagnostic imaging within a single agent, seamlessly connected and integrated by nanoparticles [86]. In recent years, advancements in nanotechnology-based approaches have paved the way for targeted therapy breakthroughs, offering a fresh approach to tackling chemo- and radioresistance. Nanoparticles can be tailored to specifically address the fundamental characteristics and mechanisms of CSCs, thereby paving the way for more efficacious treatment strategies. Nevertheless, numerous challenges persist in the widespread adoption of these formulations. With the expanding horizons of nanotechnology, the potential of nanotheranostics, a convergence of diagnostics and therapy, holds great promise [87]. For instance, consider the utilisation of RNAi-delivering nanoplatforms for targeting stem cells in CSC-focused anticancer therapy [88].

Multifunctional hybrid nanoparticles represent a burgeoning class of drug delivery carriers capable of facilitating targeted therapy at specific sites, mitigating side effects, and delivering combined therapies concurrently to combat drug resistance. Smiley et al. [89] recently explored initial CD133 labelling of nanoparticles for glioblastoma. The study demonstrated a noticeable trend in enhanced CSC elimination in vitro when compared to CD133-deficient nanoparticles. The incorporation of the PET radiotracer, 89Zr, into these multifunctional CD133-targetable nanoparticles introduces exciting possibilities for theranostic applications, allowing real-time in vivo tracking of their fate.

### 4.4. Machine Learning

In recent years, the scientific landscape for image analysis has been swiftly revolutionised by the rapid advancements in machine learning (ML) and artificial intelligence (AI). The progress in these fields has been greatly expedited by the emergence of high-performance computers [90]. Research endeavours have started to incorporate ML techniques in the characterisation of histopathological image analysis, particularly in the context of in vivo CSC imaging [91].

Most clinical AI studies are conducted on existing retrospective datasets, focusing on improving algorithm performance for both internal and external datasets. In a recent study by Yang et al. [92], a deep learning radiomic model based on MRI data was developed and validated from data collected retrospectively for 257 patients. This model exhibited an area under the curve (AUC) of 0.726 and 0.790 in two external validation cohorts. The study investigated Cytokeratin 19 (CK19), a stem cell marker associated with the progenitor subtype, which has been linked to heightened recurrence and therapy resistance in hepatocellular carcinoma.

Other investigations have explored AI-driven modelling to predict individual patient responses based on imaging data. Wei et al. [93] developed a gastric CSC-related score using a series of ML algorithms, enabling the prediction of immunotherapy response with an AUC of 0.736. Most recently, Zhou et al. [94] conducted research into novel immunofluorescence and AI-based comprehensive analyses to quantify and spatially analyse CD8+ T cells and CD133+ CSCs, aiming to predict the survival outcomes of patients with pancreatic adenocarcinoma.

## 5. Final Thoughts

Today, the research and clinical communities are still trying to elucidate the complex mechanisms behind one of the main causes of treatment failure in radiation oncology—tumour repopulation during treatment. Thirty-five years have passed since the landmark publication authored by R. Withers et al. on *The hazard of accelerated tumour clonogen repopulation during radiotherapy (Acta Oncologica 27:131)* [18], a paper that discusses accelerated tumour regrowth in head and neck carcinomas, and presents quantitative parameters for the clonogen doubling rate and the onset of repopulation during radiotherapy.

Currently, there is broad evidence towards the existence of cancer stem cells that are responsible for tumour repopulation, dissemination, and distant metastases in most solid cancers. For a successful management of most cancers, CSCs need identification through specific imaging techniques that can assist novel technologies to target and eradicate them.

Research is currently in the preclinical phase, and determining the suitability of these findings for conventional clinical application will necessitate further extensive preclinical studies and clinical trials. Recent advancements in preclinical oncology, particularly in immunology, are expected to identify novel molecular targets that may be utilised in future theranostic approaches such as those highlighted in the review. Further investigations are required in all directions of cancer stem cell research to shed more light on their complex properties and to identify the optimal therapies for their control.

## Data Availability

Data is available from the authors upon request.
